# A Safe and Simple Method for Self-Administration of Chloroform

**Published:** 1874-10

**Authors:** Wm. Walter Lane

**Affiliations:** Wilmington, N. C.


### ARTICLE VII.

### *A Safe and Simple Method for Self-administration of Chloroform.*

BY WM. WALTER LANE, M. D., WILMINGTON, N. C.

I noticed in the last Transactions of the State Medical Society of Virginia, that during a discussion on the use of chloroform in disease, Dr. Jenks spoke of his being a great sufferer from some heart trouble, and gave his method of self-administration, which struck me as not only being dangerous, but an expensive way. He says that he is in the habit of carrying to bed with him a pound bottle of chloroform one-fourth full, which he inhales until easy, or finds himself going to sleep, when he sets the bottle aside. But sometimes he becomes unexpectedly unconscious, the bottle falls from his hands, the contents emptied in bed, and he is awakened by the burning sensation imparted to the skin by the chloroform.

Having suffered some time ago with very severe attacks of hepatic colic, induced by the passage of biliary calculia, and having had occasion many times to administer this agent to myself, and often when I was alone, I thought I would offer through your Journal, to those in the habit of taking chloroform, a safer and simpler method, and much more economical than the one used by Dr. Jenks.

I take a small paper pill box filled with raw cotton, or lint, on which I pour a sufficient quantity of chloroform to saturate well, then lying on the back with the head slightly raised, with neck and chest exposed, and with a light handkerchief thrown over the box, the latter is held by one or both hands to the face in an elevated position, with the elbows suspended as it were above the chest. As soon as anæsthesia is produced, the arms, along with the hands containing box and handkerchief, fall upon the body, thereby avoiding all tendency to suffocation and excessive dose of the anæsthetic.

This manner of taking chloroform I have recently adopted, without the least unpleasant consequence, when suffering the most violent pain our frames are subject to.—*Am. Med. Weekly.*

